# Proteomic Mapping of Allergenic Proteins Reveals Key Differences Between Black Tiger Prawn (*Penaeus monodon*) and White Leg Prawn (*Litopenaeus vannamei*)

**DOI:** 10.1021/acs.jproteome.5c01289

**Published:** 2026-06-09

**Authors:** Janitha Iddagoda, Varsha V. Balu, Roni Nugraha, Renee Chin, Shaymaviswanathan Karnaneedi, Michael G. Leeming, Nicholas A. Williamson, Andreas L. Lopata, Thimo Ruethers

**Affiliations:** † Tropical Futures Institute, 208640 James Cook University, 149 Sims Drive, Singapore 387380, Singapore; ‡ Molecular Allergy Research Laboratory, Centre for Sustainable Tropical Fisheries and Aquaculture (CSTFA), College of Science and Engineering, James Cook University, Douglas, Queensland 4811, Australia; § ARC Industrial Transformation Research Hub (ITRH) for Supercharging Tropical Aquaculture through Genetic Solutions, James Cook University, Douglas, Queensland 4811, Australia; ∥ Department of Aquatic Product Technology, Faculty of Fisheries and Marine Sciences, IPB University, Bogor 16680, Indonesia; ⊥ Centre for Food and Allergy Research (CFAR), Murdoch Children’s Research Institute, 50 Flemington Road, Parkville, Victoria 3052, Australia; # Bio21 Molecular Science and Biotechnology Institute, University of Melbourne, 30 Flemington Road, Parkville, Victoria 3010, Australia; ○ Biomolecular Sequence To Function Division (BSFD), Bioinformatics Institute (BII), Agency for Science, Technology and Research (A*STAR), Singapore 138671, Singapore

**Keywords:** Allergens, Crustaceans, Seafood allergy, Food safety, Relative abundance, Differential abundance

## Abstract

Shellfish allergy is a major global health concern, with prawns representing the most common trigger. Black tiger prawn (*Penaeus monodon*, BTP) and white leg prawn (*Litopenaeus vannamei*, WLP) dominate global consumption. Here, label-free shotgun proteomics combined with in-silico allergenicity prediction was applied to identify and compare the allergen profiles of BTP and WLP. Raw prawn extracts were analyzed by LC-MS/MS, and allergens were identified and predicted using AllerCatPro. The relative abundance of allergens was assessed using iBAQ%, and the differential abundance was evaluated using LFQ intensity. Although allergens accounted for only ∼4% of identified proteins, they represented 34–38% of total protein abundance, highlighting their immunological relevance. Myosin light chain was the most abundant allergen, followed by arginine kinase, sarcoplasmic calcium-binding protein, and tropomyosin in both species. Four low-abundance proteins were identified as novel allergen candidates. Notable differences in allergen profiles between the two species were observed at the level of allergen orthologs, isoforms, and variants, with nine uniquely identified in BTP and seven in WLP. Together, these findings reveal species-specific variations with implications for improved diagnostic strategies, therapeutic approaches, and allergen detection methods for shellfish allergy.

## Graphical abstract

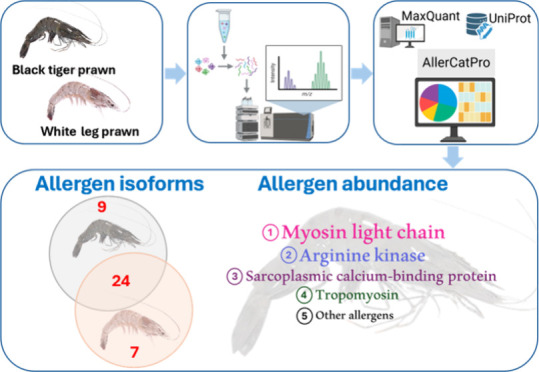

## 1 Introduction

Food allergy represents a major public health concern, affecting approximately 8% of children and 10% of adults,^1^ and shellfish is one of the nine major allergy-triggering foods worldwide.^2,3^ Shellfish allergy has a prevalence of up to 10.3% across various geographic locations and usually persists for a lifetime.^4^ Among shellfish, crustaceans, particularly prawns and shrimps are the most common triggers of allergic reactions.^5^ Prawns and shrimps are often interchangeably used terms, and in the text below “prawn” is used throughout.^6^

Of the diverse edible prawn species, Penaeid prawns sourced through aquaculture account for up to 98.2% of global farmed prawn production.^7^ Among these, black tiger prawn (BTP; *Penaeus monodon*; also known as giant tiger prawn, black tiger shrimp, or Asian tiger shrimp) and white leg prawn (WLP; *Litopenaeus/Penaeus vannamei*; also referred to as Pacific white shrimp, white shrimp or vannamei prawn) are the two most economically important species. BTPs inhabit coastal regions of Australia, Southeast Asia, South Asia, and East Africa, and account for approximately 15% of prawn aquaculture production in Asia.^8,9^ In contrast, WLP, native to the Pacific coast from Mexico to Peru, is the predominant farmed species in the Western hemisphere and contributes nearly 100% prawn aquaculture output in South America and approximately 85% in Asia.^8,10^ Together, these species account for almost 97% of the world’s farmed prawn supply, making them highly relevant allergen sources.^7^

Fourteen crustacean proteins are registered as allergens with the World Health Organisation and International Union of Immunological Societies (WHO/IUIS).^11^ Of these, nine are registered for BTP: tropomyosin (TM), arginine kinase (AK), sarcoplasmic calcium-binding protein (SCBP), myosin light chain 2 (MLC2), troponin C (TnC), triose phosphate isomerase (TPI), cytoplasmic fatty acid-binding protein (FABP), hemocyanin (HC), and glycogen phosphorylase-like protein (GPP). In contrast, only five allergens are registered for WLP: TM, AK, SCBP, MLC2 and FABP. Myosin light chain 1, troponin I, ovary development-related protein, filamin C, and mitochondrial malate dehydrogenase are registered for crustaceans other than BTP or WLP, including brine shrimp (*Artemia franciscana*), common shrimp (*Crangon crangon*), crabs, lobsters, and crayfish. In addition, pyruvate kinase, enolase, myosin heavy chain, and reticulon-like protein might be allergenic based on recent reports.^12–17^ Among all these proteins, highly conserved TM is known as the major crustacean allergen.^18^

Although BTP and WLP are closely related, emerging evidence indicates that sensitization patterns to different prawn species vary across populations, which highlights the importance of examining allergen composition at the species level.^19–21^ Studies have shown that skin prick test (SPT) extracts used for in-vivo allergy diagnosis vary considerably in their allergen composition, relative abundance, and Immunoglobulin E (IgE) antibody-binding capacity.^22,23^ Therefore, it has been suggested that region-specific allergens should be considered for more reliable diagnosis and management approaches.^24^ In this context, a detailed investigation of allergen repertoires, their relative abundance, and specific differences between BTP and WLP is essential for the development of improved diagnostic strategies, targeted therapeutic approaches, and food allergen detection methods, all of which are currently limited in the management of shellfish allergy.^25,26^

Shotgun proteomics using liquid chromatography–tandem mass spectrometry (LC-MS/MS) provides a powerful platform for characterizing allergen variability and abundance beyond the capabilities of traditional protein-based immunoassays such as ELISA, immunoblotting, or DNA-based PCR.^25,27–30^ Integrating shotgun proteomics with bioinformatics prediction tools holds strong potential for addressing current limitations in the management of shellfish allergy.^31^ AllerCatPro 2.0 has demonstrated the highest sensitivity (97%) for allergenicity prediction among the evaluated in-silico tools for seafood allergens.^32^

In this study, we combined label-free shotgun proteomics with in-silico allergenicity prediction to characterize and compare the allergen profiles of BTP and WLP. Our objectives were to (i) identify crustacean allergens in each species, (ii) quantify relative allergen abundance using intensity-based absolute quantification (iBAQ) values, (iii) compare allergen abundance between species using label-free quantification (LFQ) intensity, and (iv) evaluate species-specific differences in allergen profiles that may influence IgE antibody-binding capacity and safety risks. By mapping the allergen landscapes of two commercially important prawn species, this research provides molecular-level insights to support improved species-specific allergy diagnostics, therapeutic approaches, allergen detection in food products, and general risk assessment for shellfish-allergic populations.

## 2 Materials and Methods

### 2.1 Sampling, Protein Extraction, and Quantification

Raw BTP and WLP specimens were obtained from a commercial hatchery, with subsequent species confirmation based on distinct morphological characteristics. Both species were processed in triplicate, with each replicate corresponding to an independent animal. Deshelled muscle tissues were homogenized on ice, and proteins were extracted in 50 mM triethylammonium bicarbonate (TEAB) buffer with 5% sodium dodecyl sulfate (SDS) (1 g tissue: 6 mL buffer) overnight. Following centrifugation, protein concentration was determined using the Pierce Bicinchoninic (BCA) assay with bovine serum albumin (BSA) as the standard (Thermo Fisher Scientific).

### 2.2 Proteomics Sample Preparation

Proteomics sample preparation was performed using suspension trapping microcolumns (S-Trap, Protifi).^33^ In brief, proteins were reduced in 200 mM tris­(2-carboxyethyl) phosphine and alkylated with 1 M iodoacetamide. After acidification with phosphoric acid, samples were mixed with 100 mM TEAB in 90% methanol, loaded onto S-trap columns, and centrifuged at 4000*g* to trap/bind proteins. Trapped proteins were digested overnight at 37 °C with trypsin. The resulting peptides were sequentially eluted with 50 mM TEAB, 0.2% formic acid, and 50% acetonitrile, and freeze-dried for subsequent analysis.

### 2.3 Liquid Chromatography–Tandem Mass Spectrometry (LC-MS/MS) Analysis

Dried peptide samples were reconstituted in 3% (v/v) acetonitrile (ACN) containing 0.1% (v/v) formic acid, and 100 ng of peptides were analyzed using an Orbitrap Eclipse Tribrid system, (Thermo Fisher Scientific) coupled to an UltiMate 3000 RSLCnano UHPLC system (Thermo Fisher Scientific). Peptides were trapped on an Acclaim PepMap trapping column (Dionex-C18, 100 Å, 75 μm × 2 cm) before separation on an Acclaim PepMap RSLC analytical column (Dionex-C18, 100 Å, 75 μm × 50 cm). Mobile phases consisted of Buffer A, 0.1% (v/v) formic acid in water, and Buffer B, 100% ACN containing 0.1% (v/v) formic acid. Peptide separation was performed at a constant flow rate of 300 nL/min using the following gradient: 3% B for 6 min, 3%–26% B over 59 min, 26%–40% B over 10 min, 40%–80% B over 5 min, followed by 80% B for 5 min for column washing, and re-equilibration to 3% B for 10 min prior to the next injection. Mass spectrometric analysis was carried out using a nanoelectrospray ionization (NSI) source operated in positive ion mode, with a spray voltage of 1.9 kV, an ion transfer tube temperature of 275 °C, and an S-lens RF level of 30%. Data were acquired in data-dependent acquisition (DDA) mode using a cycle time-based method. Full MS scans were acquired in the Orbitrap over an *m*/*z* range of 375–1500 at a resolution of 120 000, with an AGC target of 400 000 and a maximum injection time of 50 ms. Precursor ions with charge states 2–7 were selected for fragmentation within a 3 s cycle time, with isotopic peaks excluded and dynamic exclusion enabled for 30 s. Selected precursors were isolated using the quadrupole mass filter (1.6 *m*/*z* isolation window) and fragmented by higher energy collisional dissociation (HCD) using a normalized collision energy of 30. MS/MS spectra were acquired in the Orbitrap at a resolution of 15 000, with an AGC target of 50 000 and a maximum injection time of 22 ms, and recorded in centroid mode.

### 2.4 Protein Identification and Quantification

LFQ was performed using MaxQuant (v2.7.5.0) against the UniProtKB database *Penaeus* (release: 2025_04, taxonomy ID: 133894, 29 248 entries). Raw files were separated into two parameter groups corresponding to BTP and WLP triplicates. Carbamidomethylation of cysteine was specified as a fixed modification, while oxidation methionine, protein N-terminal acetylation, and deamidation of asparagine and glutamine were set as variable modifications. Trypsin/P was selected as the protease, allowing up to two missed cleavages. The false discovery rate (FDR) was controlled at 1% for peptide-spectrum matches, peptides, and protein groups using the target decoy approach. LFQ intensities were generated using the MaxLFQ algorithm, which incorporates an internal delayed-normalization approach to account for systematic variations in sample preparation and LC-MS/MS performance between samples.^34^ In parallel, iBAQ values were generated to estimate the relative abundance of allergens within each sample. Data were processed with Perseus (v2.1.6.0) to remove potential contaminants, reverse hits, and proteins identified only by site. LFQ intensity values were log_2_-transformed for normal distribution prior to further statistical analysis to stabilize variance and approximate normal distribution. The processed matrix was analyzed in Microsoft Excel and GraphPad Prism (v10.6.0). For each protein group, the leading protein in the “Majority protein IDs” list was used as the representative identifier.

### 2.5 Allergen Identification and Comparative Quantification

AllerCatPro (Version 2.0) was used to predict the allergenicity of identified proteins based on their amino acid sequence similarity (% identity, linear 80 amino acid window) and 3D structural similarity (% identity, 3D epitope) to known allergens, listed in databases from WHO/IUIS, Comprehensive Protein Allergen Resource (COMPARE), Food Allergy Research and Resource Program (FARRP), UniProtKB, and Allergome.^35^ The output for each queried protein included its similarity to known allergens in the database and a resulting prediction of strong, weak, or no evidence of allergenicity. Proteins predicted to exhibit strong evidence of allergenicity against crustacean allergens in the database were selected for relative abundance and differential abundance analyses.

The relative abundance of allergens in BTP and WLP was determined by normalizing iBAQ values to iBAQ%, representing the percentage contribution of each allergen to the sum of all iBAQ values within a sample.^36,37^ Mean iBAQ% of allergens were calculated across the three biological replicates for each species. Proteins were retained if they were identified in at least two biological replicates. Where applicable, isoforms or subunits belonging to the same allergen group were aggregated to estimate total allergen abundance for each allergen group.^22^

Differential abundance analysis was performed using log_2_-transformed LFQ intensities. Proteins with missing values across all six samples were excluded. To ensure robust quantification, proteins were further filtered to retain those with at least two valid values within each species. No proteins with two missing LFQ values within a triplicate group were observed in the datasets. Missing values were then addressed using a Missing Not at Random (MNAR) imputation strategy, reflecting low-abundance proteins below the detection limit, and were imputed following the Perseus default parameter. Specifically, for proteins with exactly one missing value within a triplicate, values were drawn from a downshifted (downshift = 1.8 standard deviations) and narrowed (width = 0.3) normal distribution to simulate the MS detection limit.^38,39^ Proteins absent in one species but present in at least two replicates of the other species were retained to capture species-specific expression. Comparative analysis was conducted using multiple unpaired two-tailed *t*-tests on the 24 allergens shared between the two species, assuming unequal variances. *p*-values were adjusted using the Benjamini-Hochberg procedure to control the false discovery rate (FDR) at 1% (*q* < 0.01). Adjusted *p*-values below this threshold were considered statistically significant.^40^

## 3 Results

### 3.1 Protein Identification and Quantification

Shotgun proteomic analysis yielded comprehensive protein and allergen profiles for both prawn species (Figure 1). In total, 10 136 peptides were identified across both species, corresponding to 1758 proteins grouped into 841 protein groups. Of these, 636 proteins had valid LFQ intensities; the remaining 205 lacked complete quantitative data across all six samples and were excluded from further comparison. Among the 636 quantified proteins, 56 were unique to BTP, 209 were unique to WLP, and 371 were shared between the two species (Figure 2).

**Figure 1:**
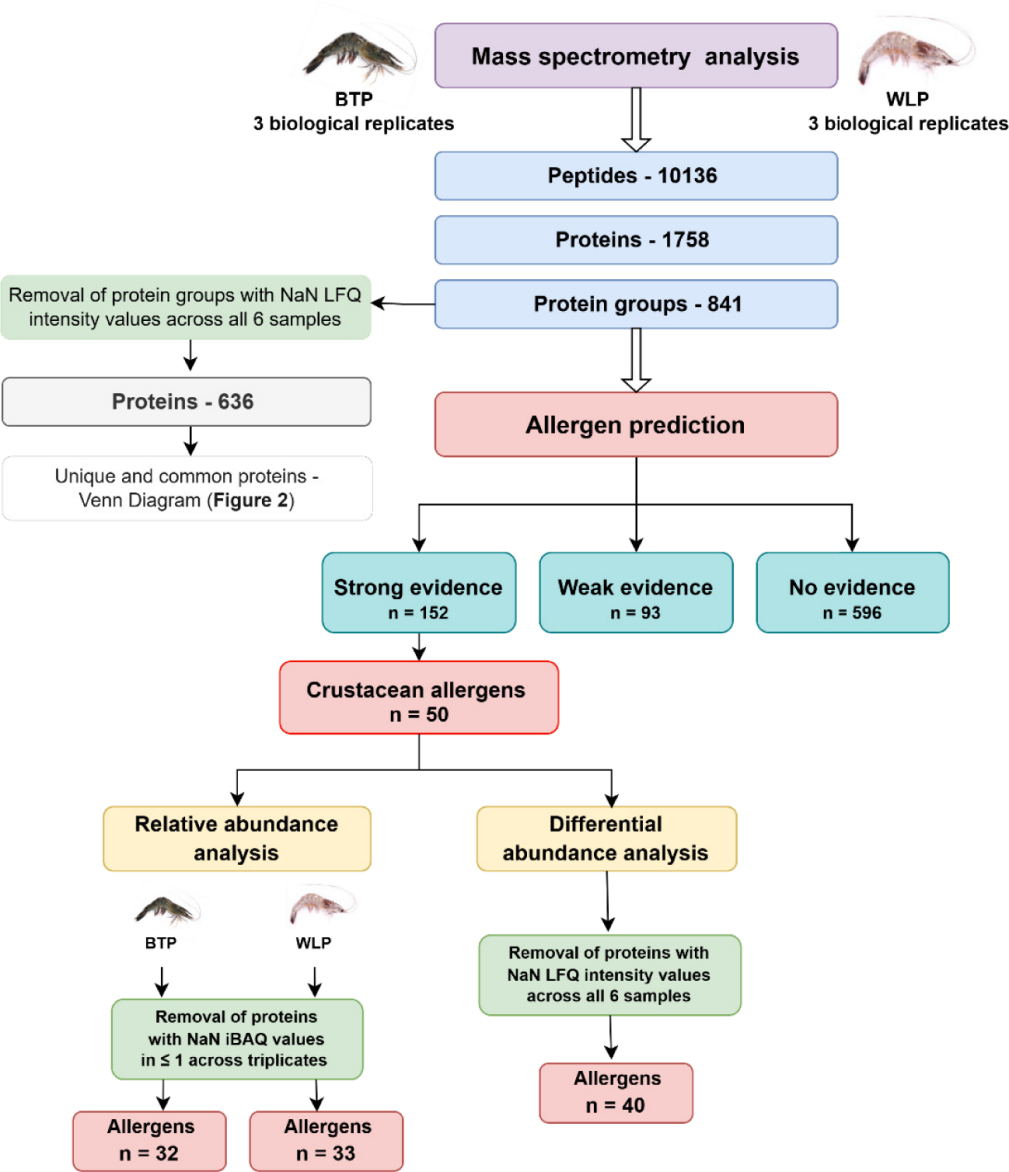
Schematic representation of protein and allergen identification. Results for peptide, protein and protein group identification, and allergen prediction are shown. The number of allergens used for relative abundance analysis of black tiger prawn (BTP) and white leg prawn (WLP), and for differential abundance analysis, is indicated.

**Figure 2:**
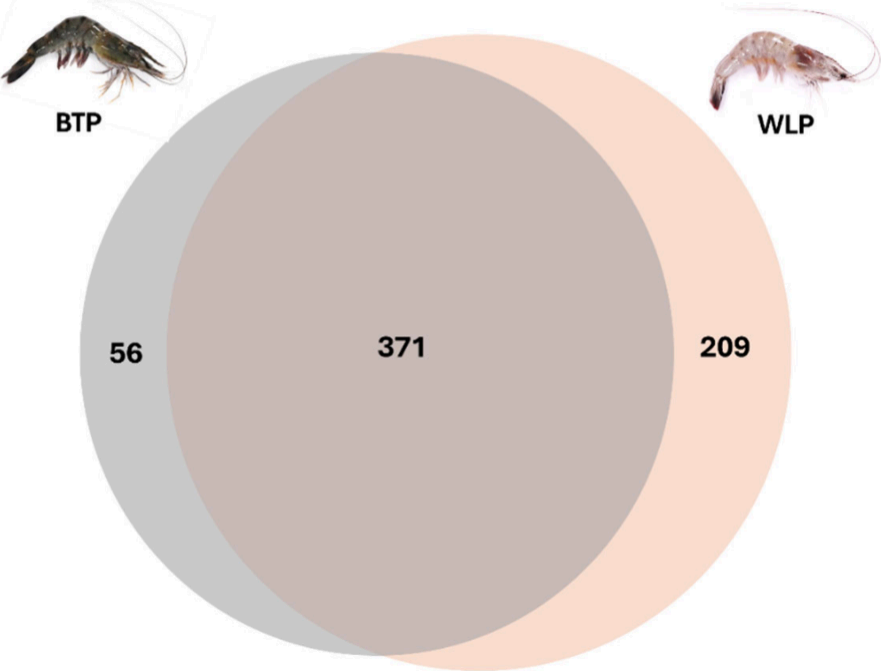
Proteins identified in black tiger prawn (BTP) and/or white leg prawn (WLP). Shared proteins are displayed in the overlapping region, while nonoverlapping segments indicate unique species-specific protein identifications.

### 3.2 Allergen Identification

Of the 841 identified proteins, AllerCatPro classified 152 as allergens with strong evidence, 93 with weak evidence, and 596 with no evidence of allergenicity. Out of 152 proteins with strong evidence for allergenicity, 50 matched known crustacean allergens. Exclusion of proteins for allergen relative abundance analysis with iBAQ values present in ≤1 replicate across triplicates resulted in 32 allergens (3.8% of the proteome) in BTP and 33 (3.9% of the proteome) in WLP. For differential abundance analysis, removal of proteins with NaN LFQ intensity values across all six samples produced a final set of 40 crustacean allergens used for statistical comparison.

Among the identified allergens, eight WHO/IUIS-registered prawn allergens which are TM, AK, MLC, SCBP, TnC, HC, TPU, and intracellular FABP were detected. Other crustacean allergens with strong predicted allergenicity (troponin I (TnI), troponin T, calcium-transporting ATPase, filamin A, and potassium channel proteins) were also identified and included in downstream analyses. Details of allergen identities and supporting evidence (best allergen hit, species, UniProt ID, SUPFAM classification, IgE prevalence, sequence identity in linear 80 aa windows and 3D epitope matching, and gene ontology annotations) are summarized in Table S1 in the Supporting Information.

### 3.3 Relative Abundance of Allergens

The total relative abundance of identified allergens (iBAQ%) was 38.16% in BTP and 34.56% in WLP (Figure 3). In BTP, MLC was the most abundant allergen (11.01%), followed by AK (8.87%), SCBP (7.19%), and TM (6.66%). The troponin complex (troponin I, C, and T) collectively accounted for 2.85% of total allergen abundance. Calcium-transporting ATPase, HC, TPI, and filamin A, demonstrated abundances ranging from 0.10% to 0.60%. FABP (0.01%) and potassium channel protein (0.001%) were the least abundant allergens (Figure 3A).

**Figure 3:**
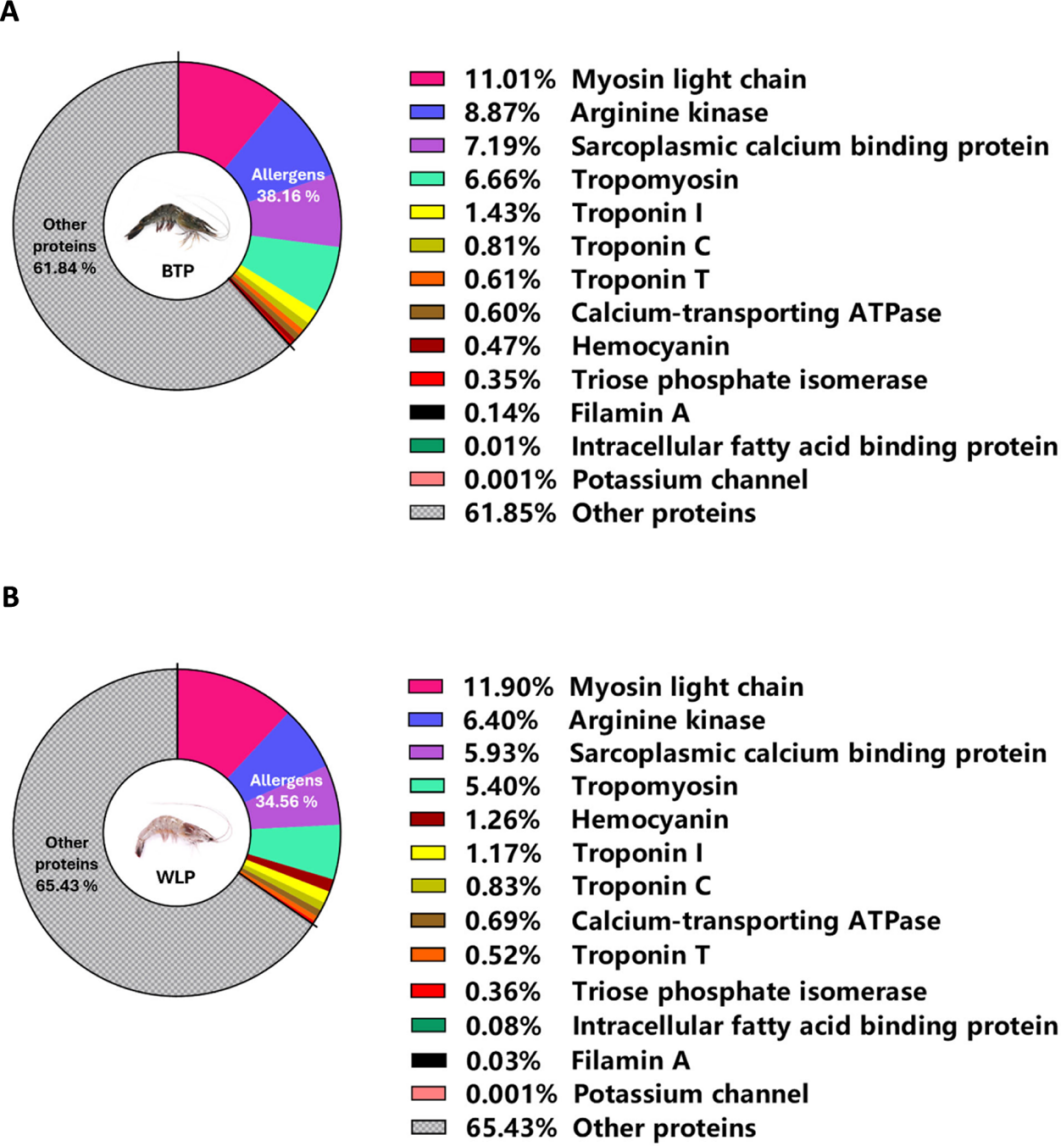
Relative abundance of allergens in black tiger prawn (BTP) (A) and white leg prawn (WLP) (B) expressed as iBAQ%. For allergen groups containing multiple isoforms or subunits, iBAQ% values were summed to obtain the total group protein abundance. Values represent the mean across three biological replicates per species. The allergens have been ranked by iBAQ% from highest to lowest for BTP and WLP, respectively.

In WLP, MLC was similarly the most abundant allergen (11.90%), followed by AK (6.40%), SCBP (5.93%), and TM (5.40%). The troponin complex accounted for 2.52% of the total allergen pool, and HC for 1.26%. Calcium-transporting ATPase (0.69%) and TPI (0.36%) showed moderate abundance, whereas FABP (0.08%) and filamin A (0.03%) were less abundant. Potassium channel protein remained the least abundant allergen (0.001%) (Figure 3B).

### 3.4 Differential Abundance of Allergens

LFQ intensity-based comparison of the 40 quantified allergens revealed significant species-specific differences (Figure 4A). Nine allergens were detected exclusively in BTP: SCBP (H7CHW2), TnI (D2SR43), and seven HC identities (A0A3R7NPL9, A0A3R7P7W3, B9VR33, G1AP69, Q95 V28, B0L612, S5ZHH2). Seven allergens were uniquely present in WLP: AK (Q004B5), MLC (A0A3R7M961), TnC (G8H4B7), and four HC identities (A0A059TFW7, A0A088MK65, A0A3R7NPL9, A0A3R7PUZ2). Differential abundance analysis of allergens shared between both species showed that HC (A0A059TEW9) and intracellular FABP (Q1KS35) were significantly more abundant in WLP, while the remaining 22 allergens were expressed at similar levels in both BTP and WLP (Figure 4B).

**Figure 4:**
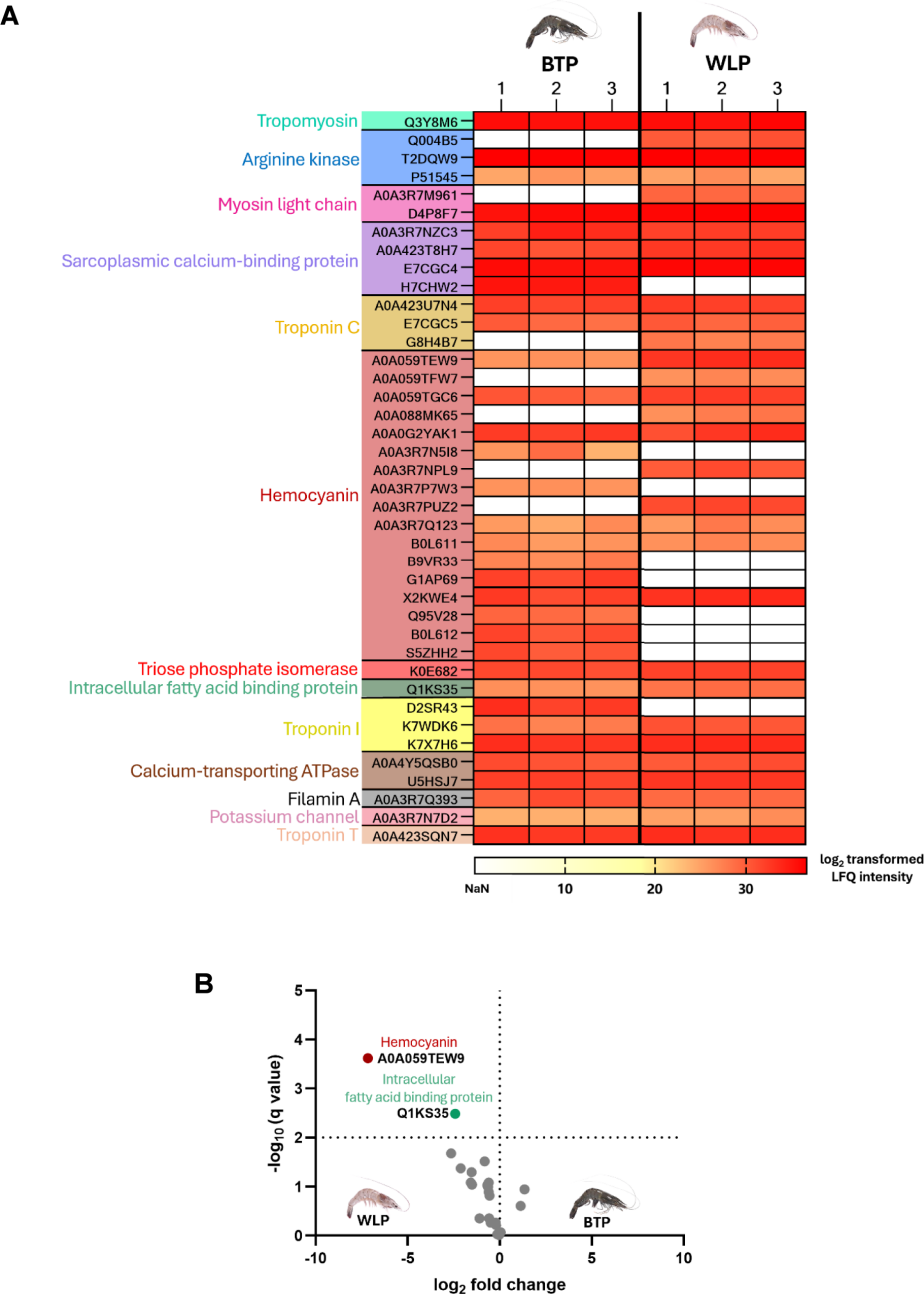
Differential abundance of allergens in black tiger prawn (BTP) and white leg prawn (WLP). (A) Heat map of allergen abundance in BTP and WLP generated using log_2_-transformed LFQ intensities. Allergen identifiers are color-coded by allergen group. White-coded entries in the matrix indicate missing values (LFQ intensity = NaN), highlighting the uniquely detected allergens across species. Numbers 1 to 3 represent biological replicates of each species. (B) Differential abundance of allergens shared between the two species. Log_2_ fold change (BTP vs WLP) is shown on the *x*-axis and – log_10_(*q* value) on the *y*-axis. Statistical significance was assessed using multiple unpaired *t*-tests with Benjamini–Hochberg FDR correction (FDR = 1%; *q* < 0.01). Significantly differentially expressed allergens are highlighted using their corresponding allergen-group colors.

## 4 Discussion

In this study, we evaluated the allergen profiles of the two most commercially important prawn species, BTP and WLP, focusing on differential allergen abundances. Although only ∼4% of identified proteins were allergens, they accounted for 34%–38% of total protein abundance, emphasizing the high allergenic potential of prawns.

Of the identified allergens, MLC, AK, SCBP, TM, TnC, TnI, HC, TPI, and FABP were among those registered with the WHO/IUIS.^11^ Although TM is the major crustacean allergen, its relative abundance was surpassed by AK, MLC, and SCBP in both species. Studies from Australia,^41^ Austria,^42^ China,^13^ Hong Kong,^12^ and Japan^21^ have shown that ∼50% of patients with a history of crustacean allergy do not react to TM in diagnostic immunoassays. Therefore, TM is not a universally accurate marker for diagnosing crustacean allergy, emphasizing the urgency of identifying the most accurate allergens as diagnostic markers for different geographical regions. Sensitization to MLC, SCBP, and AK, ranging from ∼10% to 35%, has been reported in Australia, Austria, and Hong Kong. In contrast, TnC, with relative abundance ∼5 times lower than TM, has been identified to sensitize 50% of shellfish-allergic individuals in Hong Kong and Thailand^12^ but only 11% in Austria.^12^ TnI, which was previously identified as a potential prawn allergen by de novo transcriptomic analyses, is a known allergen in crayfish (*Pontastacus leptodactylus*) but not a clinically confirmed allergen in prawns.^11,43^ There have been reports of other low-abundance allergens from east and southeast Asia: TPI (19.10% in China),^44^ FABP (39.5% in Thailand),^12^ and HC (24% in Australia).^41^ In Spain, HC of WLP was previously reported to be the cause of anaphylaxis in three patients.^45^

In addition to the WHO/IUIS-registered allergens, other proteins with comparatively much lower relative abundance, such as troponin T, calcium-transporting ATPase, filamin A, and potassium channel, were identified, and are likely to be novel allergens. Calcium-transporting ATPase (SERCA; smooth endoplasmic reticulum Ca^2+^-ATPase) has previously been identified as an IgE-binding protein in snow crab (*Chionoecetes opilio*), and the Allergome database reports it as an allergen (Chi o SERCA).^46,47^ Troponin T has not been reported as a crustacean allergen in earlier studies, and prawn troponin T showed high sequence and structural similarity to Pon I 7, the WHO/IUIS-registered TnI allergen from crayfish. Similarly, although filamin A has not previously been described as a crustacean allergen, there is high similarity to Scy p 9, a WHO/IUIS-registered filamin C allergen from mud crab (*Scylla paramamosain*).^11^ Notably, filamin A was approximately 5-fold more abundant in BTP than in WLP, suggesting potential clinical relevance in species-specific allergic reactions. Potassium channel proteins have not been identified as allergens to date. However, there is a similarity between the potassium channel and Pen m 6 (TnC) from BTP. Given the comparatively low abundance of these identified potential allergens, conventional immunoassays used in clinical diagnostics may lack sufficient sensitivity to detect IgE binding to them.

Allergic responses to the same allergen source can vary depending on its allergenic protein profile and the abundance of individual allergens, as exposure levels and immune recognition are influenced by allergen abundance.^48^ Therefore, considering the abundance of allergenic proteins during the development of management strategies is clinically important. The abundance of allergens in BTP has been shown to vary across habitats, highlighting natural biological diversity within species.^49^ Moreover, the diversity of allergens and their abundance in different body parts of crustaceans have been demonstrated.^50^ A previous study on occupational asthma in northern shrimp (*Pandalus borealis*) processing plants reported aerosolized protein concentrations of 375 and 480 ng/m^3^ for TM and AK, respectively, corresponding to a TM/AK ratio of approximately 3:4.^51^ Notably, a comparable TM/AK abundance ratio was observed in our study, suggesting consistency in the relative abundance of these major crustacean allergens across different crustacean species and exposure contexts.

However, it is important to distinguish between allergen abundance, sensitization, and diagnostic relevance. Sensitization relevance refers to an allergen’s capacity to induce an IgE response, whereas diagnostic relevance refers to its ability to predict clinical allergy.^52^ Sensitization can be assessed through IgE-binding assays; however, high allergen abundance does not necessarily correspond to high IgE-binding or diagnostic relevance.^48^ While allergen abundance may influence exposure levels, allergenicity is also determined by intrinsic properties such as protein stability and the presence and accessibility of conformational and linear IgE-binding epitopes.^53^ In addition, the allergen dose can influence immune polarization through the kinetics of major histocompatibility complex class II (MHC II) presentation.^54^ Higher antigen exposure may increase the formation of peptide-MHC II complexes and promote T helper 1 (Th1) or regulatory responses, including the production of interferon-γ (IFN-γ) and interleukin-10 (IL-10).^55^ This, in turn, can support the generation of blocking antibodies, such as IgG and IgG4, and counterbalance T helper 2 (Th2)-mediated allergic responses.^55^ In contrast, lower antigen (allergen) exposure is often associated with enhanced interleukin-4 (IL-4) production and Th2-dominated responses leading to allergic responses.^53–55^ Accordingly, low-abundance allergens could contribute to sensitization and therefore represent candidates for inclusion in future diagnostic strategies, followed by clinical validation using functional assays such as basophil activation tests (BAT) or SPT using recombinant allergens.

Numerous isoforms, variants, and/or subunits of the allergens were identified with significant differences in their abundance between BTP and WLP. TM was expressed in both species with no significant difference, confirming conserved sequence and structural similarities across crustaceans. Of the three identified AK orthologs, the AK derived from *L. vannamei* (WLP) was identified only in WLP. Similarly, among the two MLC orthologs, the MLC derived from *L. vannamei* was found only in BTP. Of the four SCBP orthologs and isoforms, a SCBP isoform originating from *P. monodon* was detected only in BTP. A wide range of distinct and common HC isoforms and subunits was identified across species. A total of seven and four of the 17 HC identities, which included isoforms, variants, and subunits of different orthologs derived from *L. vannamei* (12), *P. monodon* (2), *P. japonicus* (2), and *P. merguiensis* (1), were present exclusively in BTP and WLP, respectively. Of the six HC identities detected in both species, only one isoform (*L. vannamei*) was significantly more abundant in WLP compared to BTP. HC isoform complexity in BTP, with at least 12 isoforms, has been shown in previous research.^56^ One of the three TnC orthologs and isoforms, derived from *L. vannamei* was found exclusively in WLP. Among the orthologs and isoforms of the other allergens, FABP originally was detected in both prawns, with significantly higher abundance in WLP.^32^ Using the Allermatch tool for allergen prediction, Srisomsap et al. recently also identified enolase, HC (subunit L2), paramyosin, and SCBP (α-A and α-B chains) as potential allergens in fresh and powdered WLP, suggesting that these proteins could be used to distinguish between sensitization to krill and WLP.^57^ However, since our study specifically focused on allergens with strong similarity to crustacean allergens, enolase, identified with strong similarity to insect enolases, and paramyosin, identified with strong similarity to paramyosin from insects, molluscs, and nematodes (*Anisakis simplex*), were not included in the comparison.

Species-specific reactions to BTP and WLP during oral food challenges in shellfish-allergic individuals have been reported, further emphasizing the different clinical reactivity to BTP and WLP.^58^ In line with these findings, we suggest that the unique allergens identified in BTP and WLP in our study could serve as candidates for the development of species-specific allergy diagnostic tests, whereas allergens similarly abundant across both species may be suitable for universal diagnostic or detection tests applicable to both BTP and WLP. TM, in particular, remains a key diagnostic and detection marker for prawns in regions where sensitization to this allergen predominates (e.g., in USA, Brazil, Spain);^59,60^ however, its highly conserved characteristics and low species specificity limit its use as a discriminative marker for BTP or WLP, and potentially other prawn species. Furthermore, mass spectrometry enables targeted detection and quantification of proteotypic peptides and can facilitate the development of species-specific allergen panels integrated with IgE-binding.^61^ Integration of MS-based workflows with immunological assays has the potential to enhance the identification of clinically relevant specific allergens, including low-abundance allergens that may otherwise be overlooked.

When combined, our findings can enhance BTP and WLP allergy diagnosis, treatment, and allergen detection techniques. Currently, in-vivo diagnostic methods, such as SPT, are available both commercially and as in-house preparations; however, they vary widely in the prawn species used, allergen composition, relative allergen abundance, and extraction protocols.^22,23^ This variability limits diagnostic specificity, sensitivity, and standardization.^24^ Most in-vitro diagnostics strongly rely on allergen mixtures rather than specific components.^62^ Component resolved diagnostics (CRD), which use specific recombinant allergens, are currently restricted to a limited number of BTP allergens: TM, AK, MLC, and SCBP, while no CRD for WLP are available.^63,64^ This lack of specific components compromises diagnostic accuracy. Similarly, allergen immunotherapy approaches for prawn allergy, including oral and sublingual immunotherapy, rely on nonstandardized prawn extracts.^24,65^ The use of recombinant allergens has been proposed as a more targeted strategy, with the additional potential to engineer hypoallergenic variants that reduce the risk of adverse reactions while maintaining immunogenicity.^65^ Finally, existing food allergen detection methods predominantly target TM because of its high stability.^66^ However, our data indicate that other allergens, such as heat-stable MLC and SCBP, are also highly abundant in both BTP and WLP. Importantly, mass spectrometry itself represents a highly sensitive and specific approach for prawn allergen detection compared to conventional allergen detection assays such as ELISA, enabling simultaneous identification and quantification of multiple allergens, including isoforms and low-abundance allergens.^25,30,67^

## 5 Conclusion

In conclusion, this study provides the first comprehensive, quantitative comparison of the allergen repertoires of the two most commercially important prawn species, BTP and WLP, revealing allergen diversity and substantial differences in allergen abundances. Although allergens represent only a small fraction of the total proteome (∼4%), their high contribution to total protein abundance (34%–38%) highlights their immunological relevance. Our findings suggest that allergen abundance alone does not predict clinical sensitization, and that less-abundant allergens and isoallergens may play important roles in species-specific allergic responses. The identification of differentially expressed allergen orthologs, isoforms, variants, and subunits between BTP and WLP highlights the importance of moving beyond single-marker approaches, such as TM, toward a more comprehensive, species-specific system for allergy diagnosis and management.

By integrating quantitative proteomics with in-silico allergenicity prediction, this work highlights candidate allergens that may improve the specificity and sensitivity of diagnostic tests, CRDs, and support the development of targeted and safer immunotherapeutic strategies. Furthermore, the identification of highly abundant allergens beyond TM provides candidates to enhance food allergen detection. Finally, the findings advance our understanding of prawn allergen diversity and provide a foundation for future clinical allergen validation studies to improve the diagnosis and management of shellfish allergy.

## Supplementary Material



## Data Availability

The mass spectrometry proteomics data have been deposited to the ProteomeXchange Consortium via the PRIDE partner repository with the dataset identifier PXD072307.

## Notes

Table S1: Crustacean allergens identified by label-free quantification in black tiger prawn (BTP) and white leg prawn (WLP) with strong evidence of allergenicity predicted by AllerCatPro (PDF)

## References

[1] Bartha I., Almulhem N., Santos A. F. (2024). Feast for thought: A comprehensive
review of food allergy 2021–2023. J.
Allergy Clin. Immunol..

[2] Baker M. G., Wong L. S. Y., Konstantinou G. N., Nowak-Wegrzyn A. (2025). Food allergy
endotypes revisited. J. Allergy Clin. Immunol..

[3] Su B. B., Blackmon W., Xu C. (2024). Diagnosis and management
of shrimp allergy. Front. Allergy.

[4] Wang H. T., Warren C. M., Gupta R. S., Davis C. M. (2020). Prevalence and Characteristics
of Shellfish Allergy in the Pediatric Population of the United States. J. Allergy Clin. Immunol.: In Practice.

[5] Chen, J. ; Zhang, Q. ; Ying, Y. ; Zhang, X. ; Qu, C. Prevalence of shrimp allergy: a meta-analysis based on different diagnostic methods. Front. Allergy 2025, 6, 10.3389/falgy.2025.1635274. PMC12434110 40958988

[6] Chan, T. Shrimps and prawns. In FAO Species Identification Guide for Fishery Purposes. The Living Marine Resources of the Western Central Pacific; Carpenter, K. E. , Niem, V. H. , Eds.; Food and Agriculture Organization of the United Nations, Rome, 1998; p 851. Available via the Internet at: https://www.fao.org/4/w7192e/w7192e13.pdf (accessed December 16, 2025).

[7] Ren S., Yáñez J. M., Perez-Enriquez R. (2025). 100 Years of Penaeid Domestication and Meta-Analysis
of Breeding
Traits. Rev. Fish. Sci. Aquacult..

[8] Lelieveld, H. ; Keener, L. ; Martin-Belloso, O. ; Braun, S. ; McMahon, H. ; Astley, S. ; Prakash, V. , Science-Based Harmonization of Regulations for the Safety of Traditional and Ethnic Foods. In Regulating Safety of Traditional and Ethnic Foods, 2016; pp 481–485, 10.1016/B978-0-12-800605-4.00025-6.

[9] FAO. Penaeus monodon Fabricius. Available via the Internet at: https://www.fao.org/fishery/docs/CDrom/aquaculture/I1129m/file/en/en_gianttigerprawn.htm (accessed December 10, 2025).

[10] FAO. Penaeus vannamei. Available via the Internet at: https://www.fao.org/fishery/docs/CDrom/aquaculture/I1129m/file/en/en_whitelegshrimp.htm (accessed December 10, 2025).

[11] WHO/IUIS Allergen Nomenclature Home Page. Available via the Internet at: https://allergen.org/ (accessed December 11, 2025).

[12] Wai C. Y.
Y., Leung N. Y. H., Leung A. S. Y. (2022). Comprehending the allergen
repertoire of shrimp for precision molecular diagnosis of shrimp allergy. Allergy.

[13] Yang Y., Liu H., Zeng W. (2021). Characterization and epitope prediction of
phosphopyruvate hydratase from *Penaeus monodon* (black
tiger shrimp). J. Food Sci..

[14] Wu C. C., Lee C. H., Tyan Y. C., Huang E. S., Yu W. T., Yu H. S. (2019). Identification of
pyruvate kinase 2 as a possible crab allergen and
analysis of allergenic proteins in crabs consumed in Taiwan. Food Chem..

[15] Khanaruksombat S., Srisomsap C., Chokchaichamnankit D., Punyarit P., Phiriyangkul P. (2014). Identification
of a novel allergen from muscle and various organs in banana shrimp
(*Fenneropenaeus merguiensis*). Ann. Allergy, Asthma Immunol..

[16] Li S., Chu K. H., Wai C. Y. Y. (2023). Genomics
of Shrimp Allergens and
Beyond. Genes.

[17] Lee C. H., Wu C. C., Tyan Y. C., Yu W. T., Huang E. S., Yu H. S. (2018). Identification of
pyruvate kinase as a novel allergen in whiteleg
shrimp (*Litopenaeus vannamei*) by specific-IgE present
in patients with shrimp allergy. Food Chem..

[18] Ruethers T., Taki A. C., Johnston E. B. (2018). Seafood allergy: A comprehensive
review of fish and shellfish allergens. Mol.
Immunol..

[19] Anvari S., Brunner S., Tuano K. S. (2022). Similar IgE binding
patterns in Gulf of Mexico and Southeast Asian shrimp species in US
shrimp allergic patients. Allergy.

[20] Karnaneedi S., Anvari S., Brunner S. (2025). Shrimp Allergy—Distinct
Allergen Sensitization Profiles Between Intercontinental Cohorts. Allergy.

[21] Tsedendorj O., Chinuki Y., Ueda K., Kohno K., Adachi A., Morita E. (2018). Tropomyosin is a minor
but distinct allergen in patients
with shrimp allergies in Japan. J. Cutaneous
Immunol. Allergy.

[22] Ruethers T., Johnston E. B., Karnaneedi S. (2023). Commercial shellfish
skin prick test extracts show critical variability in allergen repertoire. Allergy.

[23] Asero R., Scala E., Villalta D. (2017). Shrimp allergy: Analysis
of commercially available extracts for in-vivo diagnosis short title:
Analysis of commercial shrimp extracts for SPT. J. Invest. Allergology Clin. Immunol..

[24] Valenta R., Karaulov A., Niederberger V. (2018). Allergen Extracts for
In Vivo Diagnosis and Treatment of Allergy: Is There a Future?. J. Allergy Clin. Immunol.: In Practice.

[25] Carrera M., Pazos M., Gasset M. (2020). Proteomics-based
methodologies for
the detection and quantification of seafood allergens. Foods.

[26] Davis C. M., Gupta R. S., Aktas O. N., Diaz V., Kamath S. D., Lopata A. L. (2020). Clinical Management of Seafood Allergy. J. Allergy Clin. Immunol.: In Practice..

[27] Bianco, M. ; Ventura, G. ; Calvano, C. D. ; Losito, I. ; Cataldi, T. R. I. Food allergen detection by mass spectrometry: From common to novel protein ingredients. Proteomics 2023, 23 (23–24), 10.1002/pmic.202200427. 37691088

[28] Kim K., Kim Y., Lee H. (2023). Discovery,
verification, and validation of walnut protein
marker peptides using LC-MS approaches. Food
Chem..

[29] Kang W., Zhang J., Li H. (2022). Quantification
of major allergens
in peach based on shotgun proteomics using liquid chromatography-tandem
mass spectrometry. LWT.

[30] Koeberl M., Clarke D., Lopata A. L. (2014). Next generation
of food allergen
quantification using mass spectrometric systems. J. Proteome Res..

[31] Krutz, N. L. ; Kimber, I. ; Winget, J. , Identification and semi-quantification of protein allergens in complex mixtures using proteomic and AllerCatPro 2.0 bioinformatic analyses: a proof-of-concept investigation. J. Immunotoxicol. 2024, 21 (1), 10.1080/1547691X.2024.2305452. 38291955

[32] Limviphuvadh V., Ruethers T., Nguyen M. N. (2025). Fish
isoallergens and
variants: database compilation, in silico allergenicity prediction
challenges, and epitope-based threshold optimization. Front. Bioinform..

[33] Elinger D., Gabashvili A., Levin Y. (2019). Suspension Trapping (S-Trap) Is Compatible
with Typical Protein Extraction Buffers and Detergents for Bottom-Up
Proteomics. J. Proteome Res..

[34] Cox J., Hein M. Y., Luber C. A., Paron I., Nagaraj N., Mann M. (2014). Accurate proteome-wide
label-free quantification by delayed normalization
and maximal peptide ratio extraction, termed MaxLFQ. Mol. Cell. Proteomics.

[35] Nguyen M. N., Krutz N. L., Limviphuvadh V., Lopata A. L., Gerberick G. F., Maurer-Stroh S. (2022). AllerCatPro
2.0: a web server for predicting protein
allergenicity potential. Nucleic Acids Res..

[36] Alvarenga P. H., Alves e Silva T. L., Suzuki M. (2024). Comprehensive Proteomics
Analysis of the Hemolymph Composition of Sugar-Fed *Aedes aegypti* Female and Male Mosquitoes. J. Proteome Res..

[37] Lim X. S., Lin Q., Chin R. (2025). Efficient protein extraction for assessing food allergy
risk in complex alternative protein matrices. Food Chem..

[38] Doncheva N. T., Schwämmle V., Locard-Paulet M. (2025). Understanding Data Analysis Steps
in Mass-Spectrometry-Based Proteomics Is Key to Transparent Reporting. J. Proteome Res..

[39] Tyanova S., Temu T., Sinitcyn P. (2016). The Perseus computational
platform for comprehensive analysis of (prote)­omics data. Nat. Methods.

[40] Bai M., Deng J., Dai C., Pfeuffer J., Sachsenberg T., Perez-Riverol Y. (2023). LFQ-Based Peptide and Protein Intensity Differential
Expression Analysis. J. Proteome Res..

[41] Johnston E. B., Kamath S. D., Iyer S. P. (2019). Defining specific allergens
for improved component-resolved diagnosis of shrimp allergy in adults. Mol. Immunol..

[42] Grilo J., Vollmann U., Aumayr M., Sturm G. J., Bohle B. (2022). Tropomyosin
is no accurate marker allergen for diagnosis of shrimp allergy in
Central Europe. Allergy.

[43] Karnaneedi S., Huerlimann R., Johnston E. B., Nugraha R., Ruethers T., Taki A. C., Kamath S. D., Wade N. M., Jerry D. R., Lopata A. L. (2021). Novel allergen
discovery through comprehensive de novo
transcriptomic analyses of five shrimp species. Int. J. Mol. Sci..

[44] Yang Y., Hu M. J., Jin T. C. (2019). A comprehensive analysis
of the allergenicity and IgE epitopes of myosinogen allergens in *Scylla paramamosain*. Clin. Exp. Allergy.

[45] Guillen D., Fiandor A., Del Pozo V. (2014). Anaphylaxis caused by
hemocyanin contained in shrimp cephalothorax. Ann. Allergy, Asthma Immunol..

[46] Abdel
Rahman A. M., Kamath S. D., Lopata A. L., Robinson J. J., Helleur R. J. (2011). Biomolecular characterization of allergenic proteins
in snow crab (*Chionoecetes opilio*) and de novo sequencing
of the second allergen arginine kinase using tandem mass spectrometry. J. Proteom..

[47] Allergome. Available via the Internet at: https://www.allergome.org/ (accessed December 13, 2025).

[48] Foo, A. C. Y. ; Mueller, G. A. Abundance and Stability as Common Properties of Allergens. Front. Allergy. 2021, 2, 10.3389/falgy.2021.769728. PMC8974735 35386965

[49] Dorney R. D., Johnston E. B., Karnaneedi S. (2024). Variation in Shrimp Allergens: Place
of Origin Effects on Food Safety Assessment. Int. J. Mol. Sci..

[50] Jerry E. M., Karnaneedi S., Ruethers T., Jerry D. R., Condon K., Lopata A. L. (2024). Allergen Diversity and Abundance in Different Tissues
of the Redclaw Crayfish (*Cherax quadricarinatus*). Foods.

[51] Abdel
Rahman A. M., Kamath S. D., Gagné S., Lopata A. L., Helleur R. (2013). Comprehensive proteomics approach
in characterizing and quantifying allergenic proteins from northern
shrimp: Toward better occupational asthma prevention. J. Proteome Res..

[52] Ansotegui I. J., Melioli G., Canonica G. W. (2020). IgE allergy
diagnostics and other
relevant tests in allergy, a World Allergy Organization position paper. World Allergy Org. J..

[53] Seidler C. A., Zeindl R., Fernández-Quintero M. L., Tollinger M., Liedl K. R. (2024). Allergenicity and Conformational
Diversity of Allergens. Allergies.

[54] Freier R., Dall E., Brandstetter H. (2015). Protease recognition
sites in Bet
v 1a are cryptic, explaining its slow processing relevant to its allergenicity. Sci. Rep..

[55] Hosken N. A., Shibuya K., Heath A. W., Murphy K. M., O’Garra A. (1995). The effect
of antigen dose on CD4+ T helper cell phenotype development in a T
cell receptor-alpha beta-transgenic model. J.
Exp. Med..

[56] Mendoza-Porras O., Kamath S., Harris J. O. (2020). Resolving hemocyanin
isoform complexity in haemolymph of black tiger shrimp Penaeus monodon
- implications in aquaculture, medicine and food safety. J. Proteom..

[57] Srisomsap C., Nonthawong K., Chokchaichamnankit D., Svasti J., Phiriyangkul P. (2023). Shotgun proteomics
characterization of potential allergens in dried and powdered krill
and fresh and powdered whiteleg shrimp. Food
Biosci..

[58] Thalayasingam M., Gerez I. F. A., Yap G. C. (2015). Clinical and immunochemical
profiles of food challenge proven or anaphylactic shrimp allergy in
tropical Singapore. Clin. Exp. Allergy.

[59] Pascal M., Grishina G., Yang A. C. (2015). Molecular diagnosis
of shrimp allergy: Efficiency of several allergens to predict clinical
reactivity. J. Allergy Clin. Immunol.: In Practice.

[60] Gámez C., Sánchez-García S., Ibáñez M. D. (2011). Tropomyosin IgE-positive results are a good predictor of shrimp allergy. Allergy.

[61] Li Y., Yang Y., Liu Y. (2022). Combination
of magnetic beads extraction
and ultraperformance liquid chromatography tandem mass spectrometry
detection for the clinical diagnosis of allergies. Anal. Chim. Acta.

[62] Thermo Fisher Scientific . ImmunoCAP Tests. Available via the Internet at: https://www.thermofisher.com/phadia/wo/en/our-solutions/immunocap-allergy-solutions.html (accessed December 13, 2025).

[63] Thermo Fisher Scientific . ImmunoCAP ISAC. Available via the Internet at: https://www.thermofisher.com/phadia/wo/en/our-solutions/immunocap-allergy-solutions/specific-ige-multiplex.html (accessed December 13, 2025).

[64] ALEX. Available via the Internet at: https://www.madx.com/products/alex (accessed December 13, 2025).

[65] Heidari, S. ; Ruethers, T. ; Karnaneedi, S. ; Yin, L. W. S. ; Lopata, A. L. Advances in Shellfish Allergy Therapy: From Current Approaches to Future Strategies. Clin. Rev. Allergy Immunol. 2025, 68 (1), 10.1007/s12016-025-09077-8. PMC12267385 40668267

[66] Lu Y., Zhang H., Gao H. (2024). Quantification of Allergic
Crustacean Tropomyosin Using Shared Signature Peptides in Processed
Foods with a Mass Spectrometry-Based Proteomic Strategy. J. Agric. Food Chem..

[67] Torii A., Seki Y., Sasano R. (2024). Development
of a rapid and reliable
method to simultaneously detect seven food allergens in processed
foods using LC-MS/MS. Food Chem..

