# Supplementary material for: Proteomic Mapping of Allergenic Proteins Reveals Key Differences Between Black Tiger Prawn (Penaeus monodon) and White Leg Prawn (Litopenaeus vannamei)
Source: J Proteome Res. 2026 Jun 9;25(7):3253–62. doi: 10.1021/acs.jproteome.5c01289 (PMC13339752; doi:10.1021/acs.jproteome.5c01289)
Supplement: Supplementary file 1 [file pr5c01289_si_001.pdf]

Supporting Information

Table 1: Crustacean allergens identified by label-free quantification in black tiger prawn (BTP) and white leg prawn (WLP) with strong evidence of allergenicity predicted by AllerCatPro

Predicted allergens		Evidence of allergenicity							iBAQ%		Gene ontology of predicted allergens		
Protein details (UniProt ID and species)	Protein/allergen name	Best known allergen hit name	Species	Best hit UniProt ID	Best hit SUPFAM	Best hit IgE prevalence	% identity: linear 80 aa window	% identity: 3D epitope	BTP	WLP	Biological process (BP)	Cellular component (CC)	Molecular function (MF)
**Detected only in BTP**													
**H7CHW2** ***P. monodon***	Sarcoplasmic calcium-binding protein	Pro c 4	*Procambarus clarkii* (Red swamp crayfish)	F6M208	EF-hand (SSF47473)	-	87.5	-	1.84%	-	-	-	calcium ion binding [GO:0005509]
**D2SR43** ***P. chinensis***	Troponin I	Pon l 7	*Astacus leptodactylus* (Danube crayfish)	P05547	Troponin coil-coiled subunits (SSF90250)	-	95	-	0.36%	-	muscle contraction [GO:0006936]	troponin complex [GO:0005861]	actin binding [GO:0003779]
**A0A3R7N5I8** ***P. vannamei***	Hemocyanin	Hemocyanin	*Litopenaeus vannamei* (White leg prawn)	A0A0G2YAK1	Hemocyanin, N-terminal domain (SSF48050); Di-copper centre-containing domain (SSF48056); E set domains (SSF81296)	-	100	100	0.01%	-	-	-	oxidoreductase activity [GO:0016491]
**A0A3R7P7W3** ***L. vannamei***	Hemocyanin	Lit v HC	*Litopenaeus vannamei* (White leg prawn)	A0A3R7NPL9	Hemocyanin, N-terminal domain (SSF48050); Di-copper centre-containing domain (SSF48056); E set domains (SSF81296)	-	100	100	0.002%	-	-	-	oxidoreductase activity [GO:0016491]
**B9VR33** ***P. chinensis***	Hemocyanin	Lit v HC	*Litopenaeus vannamei* (White leg prawn)	A0A059TEW9	Hemocyanin, N-terminal domain (SSF48050); Di-copper centre-containing domain (SSF48056); E set domains (SSF81296)	-	98.8	100	0.002%	-	-	-	oxidoreductase activity [GO:0016491]
**G1AP69** ***P. monodon***	Hemocyanin	Lit v HC	*Litopenaeus vannamei* (White leg prawn)	A0A059TEW9	Hemocyanin, N-terminal domain (SSF48050); Di-copper centre-containing domain (SSF48056); E	-	100	100	0.07%	-	-	-	oxidoreductase activity [GO:0016491]

					set domains (SSF81296)								
**Q95V28** ***P. monodon***	Hemocyanin	Lit v HC	*Litopenaeus vannamei* (White leg prawn)	X2KWE4	Hemocyanin, N-terminal domain (SSF48050); Di-copper centre-containing domain (SSF48056); E set domains (SSF81296)	-	100	100	0.02%	-	-	-	oxidoreductase activity [GO:0016491]
**B0L612** ***P. japonicus***	Hemocyanin subunit Y	Hemocyanin	*Penaeus merguiensis* (Banana prawn)	S5ZHH2	-	-	97.5	100	0.02%	-	-	-	oxidoreductase activity [GO:0016491]
**S5ZHH2** ***P. merguiensis***	Hemocyanin	Hemocyanin	*Penaeus merguiensis* (Banana prawn)	S5ZHH2	-	-	100	100	0.05%	-	-	-	oxidoreductase activity [GO:0016491]
**Detected only in WLP**													
**Q004B5** ***L. vannamei***	Arginine kinase	Lit v 2	*Litopenaeus vannamei* (White leg prawn)	Q004B5	Guanido kinase N-terminal domain (SSF48034); Glutamine synthetase/guanido kinase (SSF55931)	156	100	100	-	0.02%	dTDP metabolic process [GO:0046072]; phosphocreatine biosynthetic process [GO:0046314]	extracellular space [GO:0005615]	arginine binding [GO:0034618]; arginine kinase activity [GO:0004054]; ATP binding [GO:0005524]; creatine kinase activity [GO:0004111]
**A0A3R7M961** ***L. vannamei***	Myosin light chain	Cra c 5	*Crangon crangon* (Common shrimp)	D7F1Q1	EF-hand (SSF47473)	39	83.8	100	-	0.03%	-	muscle myosin complex [GO:0005859]	calcium ion binding [GO:0005509]
**G8H4B7** ***L. vannamei***	Troponin C1	Pen m 6	*Penaeus monodon* (Black tiger prawn)	E7CGC5	EF-hand (SSF47473)	-	100	100	-	0.02%	-	myosin II complex [GO:0016460]	calcium ion binding [GO:0005509]
**A0A059TFW7** ***L. vannamei***	Hemocyanin subunit L2	Lit v HC	*Litopenaeus vannamei* (White leg prawn)	A0A059TEW9	Hemocyanin, N-terminal domain (SSF48050); Di-copper centre-containing domain (SSF48056); E set domains (SSF81296)	-	100	100	-	0.002%	-	-	oxidoreductase activity [GO:0016491]
**A0A088MK65** ***L. vannamei***	Hemocyanin	Lit v HC	*Litopenaeus vannamei* (White leg prawn)	A0A059TGC6	Hemocyanin, N-terminal domain (SSF48050); Di-copper centre-containing domain (SSF48056); E set domains (SSF81296)	-	100	100	-	0.01%	-	-	oxidoreductase activity [GO:0016491]
**A0A3R7NPL9** ***L. vannamei***	Hemocyanin subunit L2	Lit v HC	*Litopenaeus vannamei* (White leg prawn)	A0A3R7MB02	Hemocyanin, N-terminal domain (SSF48050); Di-copper centre-containing domain (SSF48056); E set domains (SSF81296)	-	100	100	-	0.03%	-	-	oxidoreductase activity [GO:0016491]

**A0A3R7PUZ2** ***L. vannamei***	Hemocyanin	Lit v HC	*Litopenaeus vannamei* (White leg prawn)	A0A3R7P7W3	Hemocyanin, N-terminal domain (SSF48050); Di-copper centre-containing domain (SSF48056); E set domains (SSF81296)	-	100	100	-	0.13%	-	-	-
**Detected in both BTP and WLP**													
**Q3Y8M6** ***P. aztecus***	Tropomyosin	Pen a 1	*Penaeus aztecus* (Brown shrimp)	Q3Y8M6	-	1494	100	100	6.66%	5.40%	regulation of muscle contraction [GO:0006937]	-	IgE binding [GO:0019863]; protein homodimerization activity [GO:0042803]
**T2DQW9** ***P. monodon***	Arginine kinase	Lit v 2	*Litopenaeus vannamei* (White leg prawn)	B0FRF9	Guanido kinase N-terminal domain (SSF48034); Glutamine synthetase/guanido kinase (SSF55931)	156	100	100	8.87%	6.38%	phosphocreatine biosynthetic process [GO:0046314]	extracellular space [GO:0005615]	arginine kinase activity [GO:0004054]; ATP binding [GO:0005524]; creatine kinase activity [GO:0004111]
**P51545** ***P. japonicus***	Arginine kinase	Pen m 2	*Penaeus monodon* (Black tiger prawn)	T2DQW9	Guanido kinase N-terminal domain (SSF48034); Glutamine synthetase/guanido kinase (SSF55931)	127	100	100	0.002%	0.001%	phosphocreatine biosynthetic process [GO:0046314]	extracellular space [GO:0005615]	arginine kinase activity [GO:0004054]; ATP binding [GO:0005524]; creatine kinase activity [GO:0004111]
**D4P8F7** ***P. japonicus***	Myosin light chain	Cra c 5	*Crangon crangon* (Common shrimp)	D7F1Q1	EF-hand (SSF47473)	39	90	100	11.01%	11.86%	-	muscle myosin complex [GO:0005859]	calcium ion binding [GO:0005509]
**A0A3R7NZC3** ***P. vannamei***	Sarcoplasmic calcium-binding protein variant a	Lit v 4	*Litopenaeus vannamei* (White leg prawn)	C7A639	EF-hand (SSF47473)	-	76.2	-	0.82%	0.35%	-	-	calcium ion binding [GO:0005509]
**A0A423T8H7** ***P. vannamei***	Sarcoplasmic calcium-binding protein, beta chain	Lit v 4	*Litopenaeus vannamei* (White leg prawn)	C7A639	EF-hand (SSF47473)	-	100	-	0.19%	0.37%	-	-	calcium ion binding [GO:0005509]
**E7CGC4** ***P. monodon***	Sarcoplasmic calcium binding protein	Pen m 4	*Penaeus monodon* (Black tiger prawn)	H7CHW2	EF-hand (SSF47473)	91	100	-	4.34%	5.21%	-	-	calcium ion binding [GO:0005509]
**A0A423U7N4** ***P. vannamei***	Troponin C	Pen m 6	*Penaeus monodon* (Black tiger prawn)	E7CGC5	EF-hand (SSF47473)	-	77.5	100	0.71%	0.73%	-	myosin II complex [GO:0016460]	calcium ion binding [GO:0005509]
**E7CGC5** ***P. monodon***	Troponin C	Pen m 6	*Penaeus monodon* (Black tiger prawn)	E7CGC5	EF-hand (SSF47473)	-	100	100	0.10%	0.08%	-	myosin II complex [GO:0016460]	calcium ion binding [GO:0005509]
**A0A059TGC6** ***P. vannamei***	Hemocyanin subunit L1	Lit v HC	*Litopenaeus vannamei* (White leg prawn)	A0A059TFW7	Hemocyanin, N-terminal domain (SSF48050); Di-copper centre-containing domain (SSF48056); E set domains (SSF81296)	-	100	100	0.05%	0.25%	-	-	oxidoreductase activity [GO:0016491]
**A0A059TEW9*** ***P. vannamei***	Hemocyanin subunit L1	Lit v HC	*Litopenaeus vannamei* (White leg prawn)	A0A0G2YAK1	Hemocyanin, N-terminal domain (SSF48050); Di-	-	97.5	100	0.001%	0.01%	-	-	oxidoreductase activity [GO:0016491]

					copper centre-containing domain (SSF48056); E set domains (SSF81296)								
**B0L611** ***P. japonicus***	Hemocyanin subunit L	Lit v HC	*Litopenaeus vannamei* (White leg prawn)	A0A3R7Q123	Hemocyanin, N-terminal domain (SSF48050); Di-copper centre-containing domain (SSF48056); E set domains (SSF81296)	-	100	100	0.002%	0.002%	-	-	oxidoreductase activity [GO:0016491]
**A0A0G2YAK1** ***P. vannamei***	Hemocyanin V4	Lit v HC	*Litopenaeus vannamei* (White leg prawn)	A0A088MK65	Hemocyanin, N-terminal domain (SSF48050); Di-copper centre-containing domain (SSF48056); E set domains (SSF81296)	-	100	100	0.13%	0.11%	-	-	oxidoreductase activity [GO:0016491]
**A0A3R7Q123** ***P. vannamei***	Hemocyanin	Lit v HC	*Litopenaeus vannamei* (White leg prawn)	A0A3R7SZZ8	Hemocyanin, N-terminal domain (SSF48050); Di-copper centre-containing domain (SSF48056); E set domains (SSF81296)	-	100	100	0.002%	0.002%	-	-	oxidoreductase activity [GO:0016491]
**X2KWE4** ***P. vannamei***	Hemocyanin	Lit v HC	*Litopenaeus vannamei* (White leg prawn)	A0A059TEW9	Hemocyanin, N-terminal domain (SSF48050); Di-copper centre-containing domain (SSF48056); E set domains (SSF81296)	-	98.8	100	0.12%	0.71%	-	-	oxidoreductase activity [GO:0016491]
**K0E682** ***P. vannamei***	Triosephosphate isomerase	Scy p 8	*Scylla paramamosain* (Mud crab)	A0A1L5YRA2	Triosephosphate isomerase (TIM) (SSF51351)	-	96.2	100	0.35%	0.36%	gluconeogenesis [GO:0006094]; glyceraldehyde-3-phosphate biosynthetic process [GO:0046166]; glycerol catabolic process [GO:0019563]; glycolytic process [GO:0006096]	cytosol [GO:0005829]	IgE binding [GO:0019863]; triose-phosphate isomerase activity [GO:0004807]
**Q1KS35*** ***P. monodon***	Intracellular fatty acid binding protein	Pen m 13	*Penaeus monodon* (Black tiger prawn)	Q1KS35	Lipocalins (SSF50814)	-	100	100	0.01%	0.08%			fatty acid binding [GO:0005504]

**K7WDK6** ***P. vannamei***	Troponin I	Pon l 7	*Astacus leptodactylus* (Danube crayfish)	P05547	Troponin coil-coiled subunits (SSF90250)	-	95	-	0.02%	0.07%	muscle contraction [GO:0006936]	troponin complex [GO:0005861]	actin binding [GO:0003779]
**K7X7H6** ***P. vannamei***	Troponin I	Pon l 7	*Astacus leptodactylus* (Danube crayfish)	P05547	Troponin coil-coiled subunits (SSF90250)	-	96.2	-	1.06%	1.10%	muscle contraction [GO:0006936]	troponin complex [GO:0005861]	actin binding [GO:0003779]
**A0A423SQN7** ***P. vannamei***	Putative troponin T, skeletal muscle isoform X3	Pon l 7	*Astacus leptodactylus* (Danube crayfish)	P05547	Troponin coil-coiled subunits (SSF90250)	-	36	-	0.61%	0.52%	muscle contraction [GO:0006936]; regulation of muscle contraction [GO:0006937]; sarcomere organization [GO:0045214]	troponin complex [GO:0005861]	tropomyosin binding [GO:0005523]
**A0A4Y5QSB0** ***P. vannamei***	Calcium-transporting ATPase	Chi o SERCA	*Chionoecetes opilio* (Snow crab)	P86911	Calcium ATPase, transduction domain A (SSF81653)	-	97.5	-	0.03%	0.02%	-	sarcoplasmic reticulum membrane [GO:0033017]	ATP binding [GO:0005524]; ATP hydrolysis activity [GO:0016887]; metal ion binding [GO:0046872]; P-type calcium transporter activity [GO:0005388]
**U5HSJ7** ***P. vannamei***	Calcium-transporting ATPase	Chi o SERCA	*Chionoecetes opilio* (Snow crab)	P86911	Calcium ATPase, transduction domain A (SSF81653)	-	97.5	-	0.58%	0.67%	-	sarcoplasmic reticulum membrane [GO:0033017]	ATP binding [GO:0005524]; ATP hydrolysis activity [GO:0016887]; metal ion binding [GO:0046872]; P-type calcium transporter activity [GO:0005388]
**A0A3R7Q393** ***P. vannamei***	Putative filamin-A isoform X4	Scy p 9	*Scylla paramamosain* (Mud crab)	QFI57017	-	-	97.5	-	0.14%	0.03%	actin cytoskeleton organization [GO:0030036]	-	actin filament binding [GO:0051015]
**A0A3R7N7D2** ***P. vannamei***	Potassium channel	Pen m 6	*Penaeus monodon* (Black tiger prawn)	E7CGC5	EF-hand (SSF47473)	-	78.8	94.4	0.001%	0.001%	action potential [GO:0001508]; protein homooligomerization [GO:0051260]	organelle [GO:0043226]; voltage-gated potassium channel complex [GO:0008076]	calcium ion binding [GO:0005509]; delayed rectifier potassium channel activity [GO:0005251]

*Protein is significantly more abundant in WLP compared to BTP

All predicted allergens are grouped into three categories: detected only in BTP, detected only in WLP, and detected in both BTP and WLP. Allergen evidence was obtained from AllerCatPro (https://allercatpro.bii.a-star.edu.sg/), providing **Allergen hit name** (most similar known allergen in the AllerCatPro database), **Species** (the species from which the best-matched allergen originates), **Best-hit UniProt ID** (UniProt identifier corresponding to the best-matched allergen), **Best-hit SUPFAM** (predicted structural/functional domains associated with the allergen based on the SUPFAM database), **Best-hit IgE prevalence** (total number of individuals that have been tested for specific IgE towards the allergen in published epidemiology studies that are listed in the Allergome database), **% identity (linear 80 aa window)** (sequence identity in the most conserved 80-amino-acid region), **% identity (3D epitope)** (predicted structural similarity of conformational epitopes). The **relative abundance of allergens** (**iBAQ%**) in BTP and WLP was calculated separately for each species using intensity-based absolute quantification (iBAQ). Gene Ontology (GO) annotations (**BP**, **CC**, and **MF**) were retrieved from UniProt for each protein identity with predicted allergenicity.
